# Effects of intercropping teak with *Alpinia katsumadai* Hayata and *Amomum longiligulare* T.L. Wu on rhizosphere soil nutrients and bacterial community diversity, structure, and network

**DOI:** 10.3389/fmicb.2024.1328772

**Published:** 2024-02-19

**Authors:** Wang Xianbang, Lin Mingping, Li Kunliang, Han Qiang, Pan Dongkang, Ma Haibin, Huang Guihua

**Affiliations:** Research Institute of Tropical Forestry, Chinese Academy of Forestry, Guangzhou, China

**Keywords:** teak, intercropping, network analysis, soil bacterial community, medicinal plant

## Abstract

Teak is a precious hardwood species in tropical and subtropical regions with a long growth cycle and slow economic returns. Intercropping medicinal plants is an effective method for obtaining early returns during the growth period of teak. However, currently, we lack sufficient knowledge about the impact of intercropping on the soil microenvironment, especially on rhizosphere soil bacterial communities. We selected two medicinal plants *Alpinia katsumadai* Hayata and *Amomum longiligulare* T.L. Wu, for an intercropping experiment with teak, and the non-intercropping teak forest area was used for comparison. By collecting soil rhizosphere samples and conducting 16S rDNA sequencing and property analysis, we aimed to investigate the influence of teak intercropping on soil microbial communities. The results showed that intercropping significantly improved soil nutrients contents, such as soil organic matter, soil total potassium and soil available nitrogen, and significantly altered bacterial community structure. Co-occurrence network analysis revealed that intercropping tightened the connections of the soil bacterial network and increased its complexity (by increasing the number of nodes and the proportion of positive edges). Teak intercropping with *Amomum longiligulare* T.L. Wu resulted in tighter network connections than teak intercropping with *A. katsumadai* Hayata. Changes in the soil bacterial community structure may related to environmental factors such as total potassium content and pH. These results demonstrated that the introduction of medicinal plants exerts a significant impact on the soil bacterial community of teak, fostering the enrichment of specific bacterial taxa (such as *Firmicutes* and *Methylomirabilota*), and makes the rhizosphere bacterial network denser and more complex. This study provides valuable insights for the management of teak plantations.

## 1 Introduction

Soil microorganisms play a crucial role in promoting nutrient cycling and energy flow in ecosystems, and driving plant growth (Jacoby et al., 2017; Qiang et al., 2021). Vegetation composition can influence the structure of soil microbial communities (Grayston et al., 2001), as microorganisms typically rely on plant root exudates and litter as their primary nutrient sources (Grayston et al., 1998). Changing the vegetation composition can impact soil carbon cycling by altering the quantity and quality of litter and root exudates (Ward et al., 2009), subsequently inducing changes in the structure and function of soil microbial communities (De Deyn et al., 2008). Consequently, the simultaneous presence of diverse plant species immensely important for proper ecosystem functioning (De Deyn et al., 2009; Jiao et al., 2023).

Intercropping refers to the planting of more than two species of plants on a given piece of land, and plant-interspecific interactions can result in both positive and negative adaptive outcomes (Zhou et al., 2023). In this context, when the presence of one plant species alters the environment and increases the adaptability of neighboring species, a promoting effect is achieved (Brooker et al., 2021). Certain plants can facilitate the establishment of their neighbors by increasing soil nutrient availability (Wright et al., 2017), and this promoting effect can also occur indirectly through the regulatory activities of soil and microorganisms (Chen et al., 2020; Yu R. P. et al., 2021). Typically, intercropping enhances plant growth and improves the utilization efficiency of resources such as water and sunlight compared to monoculture planting (Esnarriaga et al., 2020; Jiao et al., 2023). A sugarcane and soybean intercropping experiment demonstrated that intercropping can increase the microbial diversity of rhizosphere soil, and this practice has also been widely employed to mitigate nitrogen loss and stabilize crop yield (Lian et al., 2019). The involvement of soil microorganisms in nutrient cycling can support microbial growth, which influences productivity and biodiversity (Rasche and Cadisch, 2013). Therefore, it is crucial to investigate the correlation between different vegetation compositions, soil properties, and microorganisms in intercropping systems for sustainable forest management.

Intercropping has gained widespread application in agriculture due to its potential to improve resource utilization (Hage-Ahmed et al., 2013; Fu et al., 2015; Wu et al., 2016; Nishioka et al., 2019). In the field of forestry, which has a long production and management cycle, intercropping has been increasingly studied for its ability to increase resource utilization. Many studies have focused on understory medicinal plants such as *Atractylodes lancea* (Li et al., 2018), *Panax notoginseng* (Kui et al., 2021), and *Polygonatum sibiricum* (Yi et al., 2016). Furthermore, analysis has shown that intercropping in forests can provide a healthy microbial environment for medicinal plants and increase their growth and yield (Wang et al., 2023). Therefore, it is crucial to choose appropriate plant species when establishing intercropping systems, and it is worthwhile to investigate the role of plant-mediated microbial community changes in different intercropping patterns.

Changes in microbial community structure are reflected not only in species compositions but also in interactions between taxa. Microorganisms usually form intricate ecological networks, and their interactions produce both positive (e.g., symbiosis) and negative (e.g., competition) impacts (Chen et al., 2018). Co-occurrence network analysis has been employed to discern the interactions within microbial communities (Banerjee et al., 2018). The mixed planting of *Cunninghamia lanceolata* and *Phoebe bournei* alters the symbiotic pattern of the soil microbial community and increases the diversity and connectivity of the soil microbial community (Ding et al., 2022). Climate warming has amplified the complexity of soil microbial community networks in grassland ecosystems (Yuan et al., 2021). However, the question of whether intercropping increases the complexity of microbial co-occurrence networks in teak plantations remains to be evaluated.

Teak is one of the more expensive tropical hardwoods, and is indigenous to Myanmar, Thailand, Laos, and India (Huang et al., 2019; Yu et al., 2021b). It was introduced to China more than 170 years ago and is now distributed throughout tropical China (Yang et al., 2020). Due to its long growth cycle and frequent exposure to biotic and abiotic stresses such as drought and low temperature, it takes a considerable amount of time to recuperate early investment costs (Yu et al., 2021a). Therefore, intercropping cash crops within forests is recommended to generate early income. Both *Alpinia katsumadai* Hayata and *Amomum longiligulare* T.L. Wu belong to the family Zingiberaceae and are commonly found in Guangdong and Hainan, China. These plants are considered traditional medicinal herbs in South China due to their high medicinal value (Lee et al., 2003; Ban et al., 2022), and they are listed in the Pharmacopeia of the People's Republic of China. Given the close correlation between soil bacterial communities and plant growth as well as nutrient uptake, exploring the microbiome disparities between teak and *A. katsumadai* Hayata and *A. longiligulare* T.L. Wu intercropping systems may offer fresh insights for future teak planting. In this study, we intercropped *A. katsumadai* Hayata and *A. longiligulare* T.L. Wu in part of a teak forest, while the non-intercropped area of the teak forest served as a control. This investigation involved three questions: (1) Does intercropping medicinal plants with teak improve soil nutrient characteristics compared to non-intercropping? (2) Does the rhizosphere microbial community structure of teak differ when teak is intercropped with medicinal plants? (3) Which intercropping pattern results in a more complex bacterial network structure?

## 2 Materials and methods

### 2.1 Experimental location

The experiment was conducted in Jianfengling, located in Hainan Province, China, which is within the tropical monsoon climate region. The site's altitude is 60 m. The area experiences an average annual temperature of 24.5°C and an average annual precipitation of 1,650 mm. The dry and wet seasons are clearly defined, with the rainy season spanning from May to October. The soil type is yellow latosol.

### 2.2 Experimental design and sampling

The teak forest is a clone established in 1982, with a row spacing of 5 m × 5 m. In 2019, two medicinal plants, *A. katsumadai* Hayata and *A. longiligulare* T.L. Wu were intercropped in part of the teak forest. A completely random block design was adopted with three replicates, and the row spacing of the plants was 1 m × 1 m. The details of the experimental design are shown in Supplementary Figure S1. Rhizosphere soil samples were collected in December 2021. After removing the topsoil along the root spreading direction of the teak trees via the S-type 5-point sampling method, the excess attached soil was removed by artificial vigorous shaking, and the rhizosphere soil was collected from the root surface with a sterile brush. We collected teak rhizosphere soil from the non-intercropped area (ck) and soil from the side of teak roots near *A. katsumadai* Hayata (C) and *A. longiligulare* T.L. Wu (S) from the intercropping area. Five samples from each test area were mixed, filtered with a 2 mm mesh and divided into two parts for soil property determination and DNA extraction. The samples used for soil property determination were naturally dried at room temperature (25–28°C) and subsequently stored. The samples used for DNA extraction were stored at −80°C.

### 2.3 Soil property analysis

A pH meter (Cole-Parmer ph700) was used to measure the pH of the soil suspension (water to soil ratio of 5:2) to represent the soil pH value, the soil organic matter (SOM) content was determined by the K_2_Cr_2_O_7_ oxidation method (Nelson and Sommers, 1982), soil total nitrogen (TN) was determined by the automatic Kjeldahl apparatus method (Lloret et al., 2005), soil total phosphorus (TP) was determined by the molybdenum blue method (Stauffer, 1983), soil total potassium (TK) was determined by the sodium hydroxide melting method (Ma et al., 2020), and soil available phosphorus (AP) was determined by the Olsen method (Recena et al., 2022). Available potassium (AK) in the soil was determined by a flame photometer (Xu Y. M. et al., 2020), and the soil available nitrogen (AN, hydrolyzable N) extracted with the KCL solution was analyzed using indophenol blue colorimetry and dual-wavelength ultraviolet spectrometry (Liu Z. Q. et al., 2020). The soil cation exchange capacity (CEC) was determined by extracting hexamminecobalt trichloride via spectrophotometry (Chen et al., 2019).

### 2.4 Soil DNA extraction and Illumina Miseq sequencing

Genomic DNA was extracted from fresh soil samples using the E.Z.N.A. soil DNA kit (Omega Biotek, Inc., USA) following the manufacturer's instructions. The extracted genomic DNA concentration and purity were measured using a NanoDrop 2000 UV–VIS spectrophotometer (Thermo Fisher Scientific, Inc., USA), and the soil DNA samples were stored at −20°C for subsequent experiments. The primers 338F (5′-ACTCCTACGGGAGGCAGCAGG-3′) and 806R (5′-GGACTACHVGGGTWTCTAAT-3′) (Xu M. P. et al., 2020) were used to amplify the V3–V4 region of the bacterial 16S rRNA gene. An 8 bp barcode sequence was added to the 5′ end of each upstream primer and downstream primer, and PCR amplification was performed using an ABI 9700 PCR thermal cycling apparatus (Applied Biosystems, Inc., USA). PCR products were assessed by 1% agarose gel electrophoresis and purified with an Encourt AMPure XP nucleic acid purification kit (Beckman Coulter, Inc., USA). 16S rRNA gene amplicon sequencing was performed on the Illumina MiSeq PE300 platform (Illumina, Inc., USA) according to the standard protocol of Allwegene Tech Co., Ltd. (Beijing, China).

### 2.5 Bioinformatics and statistical analysis

The QIIME (v1.8.0) microbial ecology software platform was used to analyze the data. Trimmomatic was used to perform quality control on the original fastq files, FLASH (v1.2.7) was utilized to splice the sequence data, and UPARSE (v7.1) software was used to cluster the purified sequence data. These sequences were grouped into operational taxonomic units (OTUs) with 97% similarity. The RDP Classifier (v2.2) was used to analyze the classification of each OTU sequence in the Sliva database (version 138). The confidence threshold was set at 0.9. Principal coordinate analysis (PCoA) using the unweighted UniFrac distance metric and nonmetric multidimensional scaling (NMDS) were carried out to visualize changes in microbial community structure, and LEfSe analysis was performed using Phython (v2.7) software (Segata et al., 2011).

Univariate analysis of variance (ANOVA) was used to investigate variations in rhizosphere soil properties, diversity indices, and relative abundances of major bacteria [normalized by log_10_(*x* + 1)]. Similarity analysis (ANOSIM) and permutation multivariate analysis of variance (PERMANOVA) were both carried out using Bray–Curtis distance and 999 permutations to statistically assess the differences among the bacterial communities. Spearman correlation analysis was carried out using R (v3.6.0) to explore the correlation between soil properties and bacterial diversity under the different intercropping systems.

A network analysis method was used to perform correlation analysis on the 50 most abundant OTUs in the soil bacterial communities (Ji et al., 2021). A co-occurrence network was established based on PCoA using the psych package in R software. Strong correlations (correlation coefficient *R* > 0.6) and significant correlations (*p* < 0.01) were determined to investigate the co-occurrence patterns of the soil bacterial communities. The Gephi interactive platform was used for network visualization and calculation of network topology parameters (Zhu et al., 2019).

## 3 Results

### 3.1 Soil characteristics

There were significant differences in soil organic matter (SOM), total nitrogen (TK), and available nitrogen (AN; *p* < 0.05, Table 1). Compared to those in the non-intercropped teak forest (ck), the SOM, TK, and AN in the rhizosphere soil samples (C and S) were significantly greater. Although the other soil characteristic indices were not significant (*p* < 0.05), their values were greater than those of the ck, and the soil pH increased in the two rhizosphere soil samples; soil pH was closer to neutral in C and S than in the ck. The soil properties also differed between the two rhizosphere soil samples, with higher SOM, TK, AK, and CEC values C samples than in S samples. This finding indicates that understory intercropping changed the soil properties.

**Table 1:** Soil characteristics of the rhizosphere soil samples.

**Soil properties**	**C**	**S**	**ck**
pH	7.1 ± 0.1 a	6.92 ± 0.06 a	6.89 ± 0.07 a
SOM (g/kg)	18.02 ± 1.16 b	17.4 ± 2.27 b	13.88 ± 1.84 a
TN (g/kg)	0.83 ± 0.09 a	0.93 ± 0.1 a	0.71 ± 0.06 a
TP (g/kg)	0.33 ± 0.02 a	0.36 ± 0.08 a	0.3 ± 0.01 a
TK (g/kg)	37.85 ± 2.83 b	35.55 ± 2.04 b	30.88 ± 3.41 a
AN (mg/kg)	61.65 ± 6.82 ab	66.73 ± 7.52 a	54.87 ± 4.78 b
AP (mg/kg)	5.01 ± 0.73 a	6.86 ± 1.44 a	4.01 ± 0.57 a
AK (mg/kg)	118.48 ± 16.54 a	114.83 ± 10.92 a	100.89 ± 15.42 a
CEC (cmol/kg)	6.51 ± 0.15 a	6.16 ± 0.43 a	5.82 ± 0.23 a

A different letter in each line represents a significant difference (ANOVA, p < 0.05). C represents teak forest intercropped with *Alpinia katsumadai* Hayata, S represents teak forest intercropped with *Amomum longiligulare* T.L. Wu, and ck represents non-intercropped teak forest.

### 3.2 Sequence summary and soil bacterial community diversity

**Figure 1:**
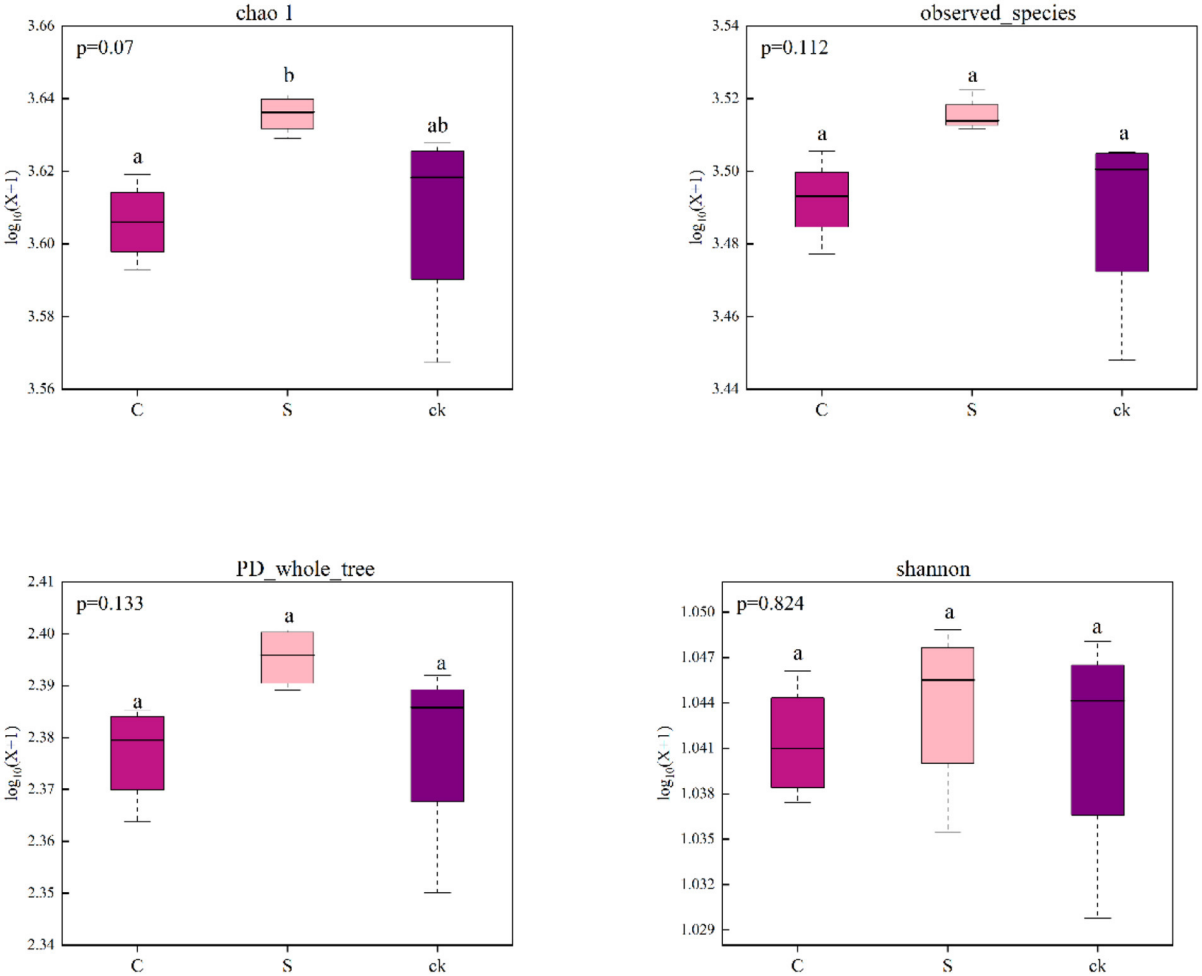
Alpha diversity indices of the bacterial communities in the soil rhizosphere samples (*n* = 4). C represents *Alpinia katsumadai* Hayata intercropped with teak forest, S represents *Amomum longiligulare* T.L. Wu intercropped with teak forest, and ck represents non-intercropped teak forest. Different letters represent significant differences between treatments.

The bacterial sequences were clustered into 5,618 OTUs, 326 of which were unique to C soil samples, 206 of which were unique to S soil samples, 262 of which were unique to ck soil samples, and 3,669 of which were shared by all the soil samples (Supplementary Figure S2). We found that there was no significant difference in the bacterial alpha diversity indices (Chao1, observed_species, PD_whole_tree and Shannon) among the different soil rhizosphere samples, indicating that understory intercropping had no significant impact on the richness or diversity of the bacterial communities (Figure 1).

### 3.3 Changes in the bacterial community composition and structure

**Figure 2:**
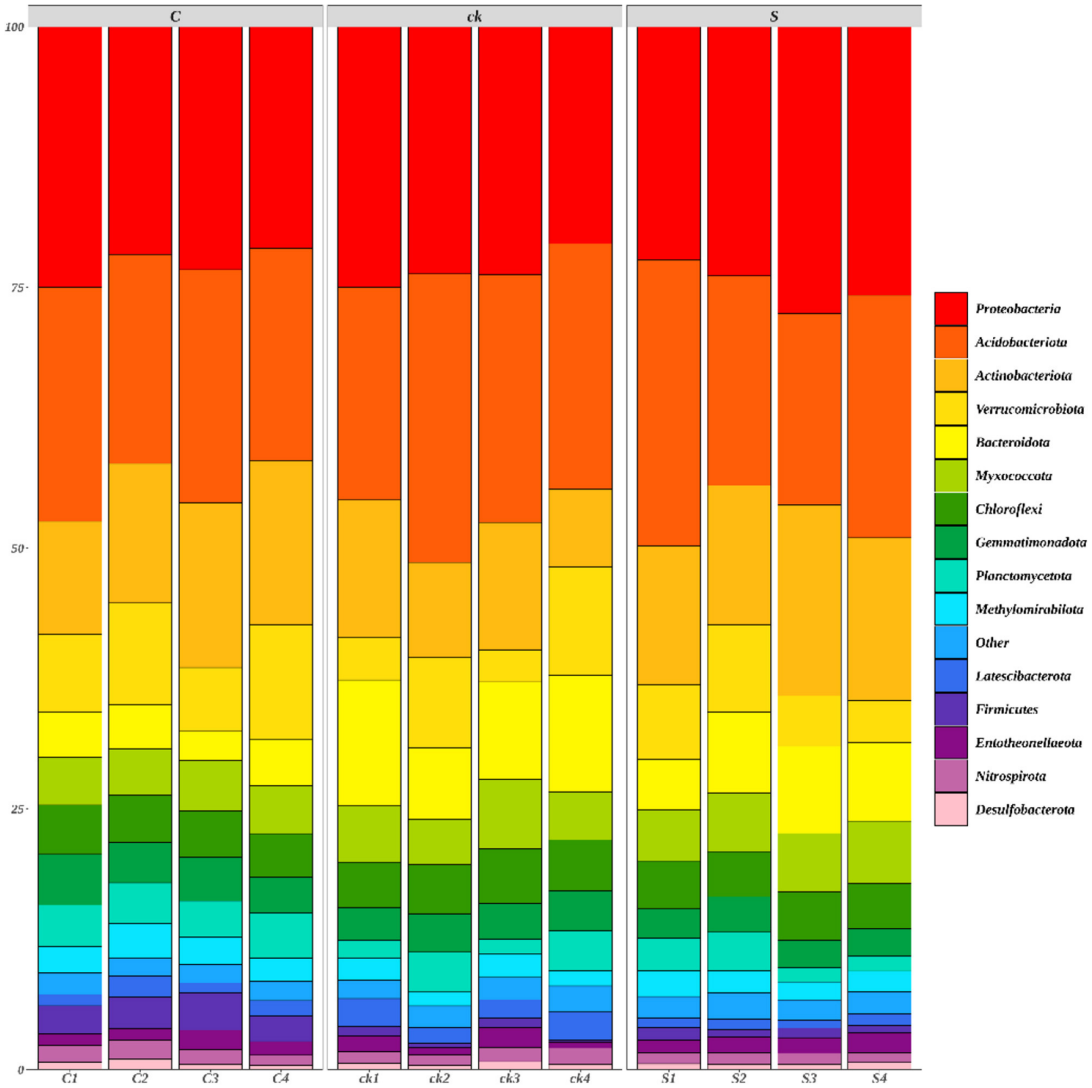
Relative abundances (>1%) of rhizosphere soil bacterial communities (phylum; *n* = 4). C represents teak forest intercropped with *Alpinia katsumadai* Hayata, S represents teak forest intercropped with *Amomum longiligulare* T.L. Wu, and ck represents non-intercropped teak forest.

A total of 42 bacterial phyla were identified across all the rhizosphere soil samples, with 15 bacterial phyla having a relative abundance >1% (Figure 2).

The relative abundances of *Actinobacteriota, Methylomirabilota* (*p* < 0.05), *Firmicutes* (*p* < 0.05) and *Entotheonellaeota* in C and S samples were greater than those in ck samples. However, for *Acidobacteriota, Bacteroidota* (*p* < 0.05), *Chloroflexi*, and *Latescibacterota* (*p* < 0.05), the relative abundances in C and S rhizosphere soil samples were lower than those in the ck samples. Additionally, the relative abundances of *Proteobacteria* and *Myxococcota* in S samples were greater than those in C and ck samples, and the relative abundance in C samples was the lowest. Moreover, significant differences in the abundances of *Bacteroidota, Gemmatimonadota, Firmicutes*, and *Nitrospirota* were observed in the soil bacterial communities of C and S samples, indicating that the different intercropping systems had an impact on the structure of the soil bacterial communities.

**Figure 3:**
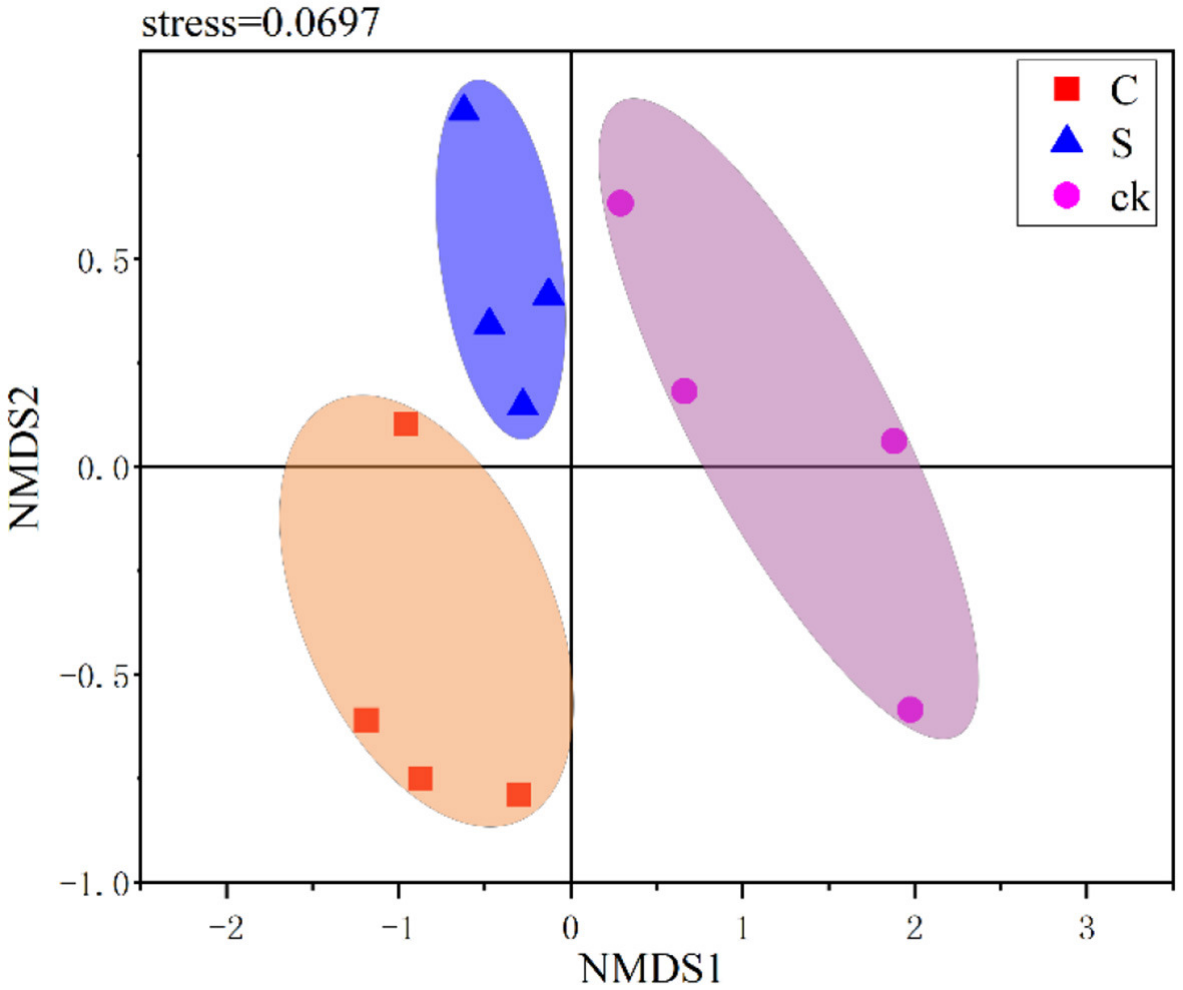
Nonmetric multidimensional scaling (NMDS) ranking of different soil rhizosphere samples. C represents teak forest intercropped with *Alpinia katsumadai* Hayata, S represents teak forest intercropped with *Amomum longiligulare* T.L. Wu, and ck represents non-intercropped teak forest.

In this study, NMDS was used to visualize the structural variations among different soil samples. As shown in Figure 3, the soil rhizosphere samples clustered into three distinct groups, and the community compositions were clearly distinct across the different samples. This indicated that there were significant variations in the bacterial community structure among the different soil rhizosphere samples. Furthermore, ANOSIM (*R* = 0.630, *p* = 0.001) and PERMANOVA (*R*^2^ = 0.402, *p* = 0.002) also confirmed significant differences in the bacterial community structure of the rhizosphere soil among the treatments.

**Figure 4:**
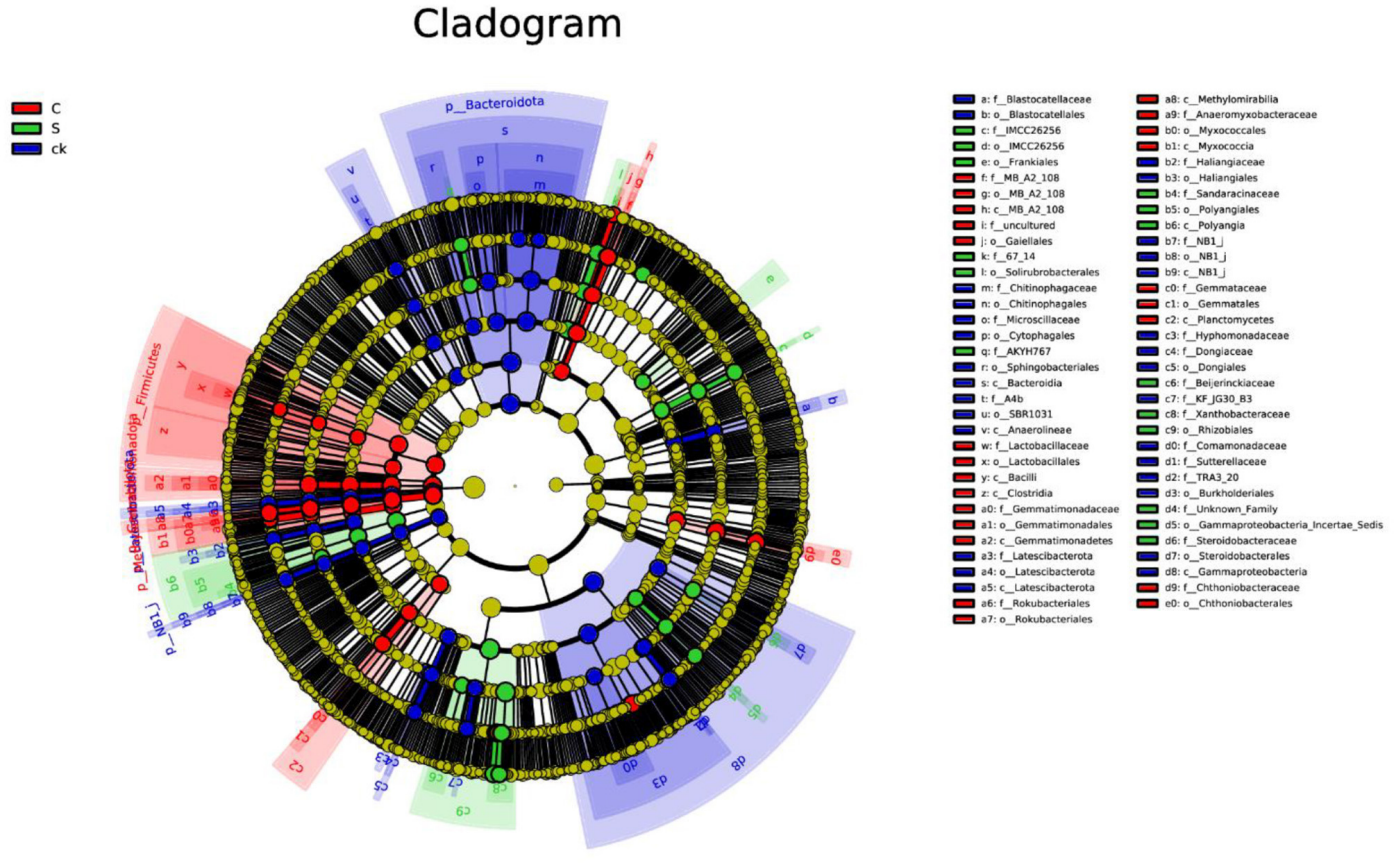
LEfSe analysis of bacterial biomarkers in rhizosphere soil samples. C represents teak forest intercropped with *Alpinia katsumadai* Hayata, S represents teak forest intercropped with *Amomum longiligulare* T.L. Wu, and ck represents non-intercropped teak forest.

Analysis of the soil rhizosphere samples revealed a total of 100 biomarkers belonging to 42 different phyla (Figure 4 and Supplementary Figure S4). Among the ck samples, 41 distinct groups were identified, with *Bacteroidota* being the most prominent. Notably, the number of biomarkers found in the C and S samples was lower than that in the ck samples. Specifically, C samples exhibited 35 abundant taxa, with *Firmicutes* being particularly enriched, while S samples had only 24 biomarkers, with *Rhizobiales* (order) having the highest relative abundance among them.

### 3.4 Co-occurrence network of soil bacterial communities

**Figure 5:**
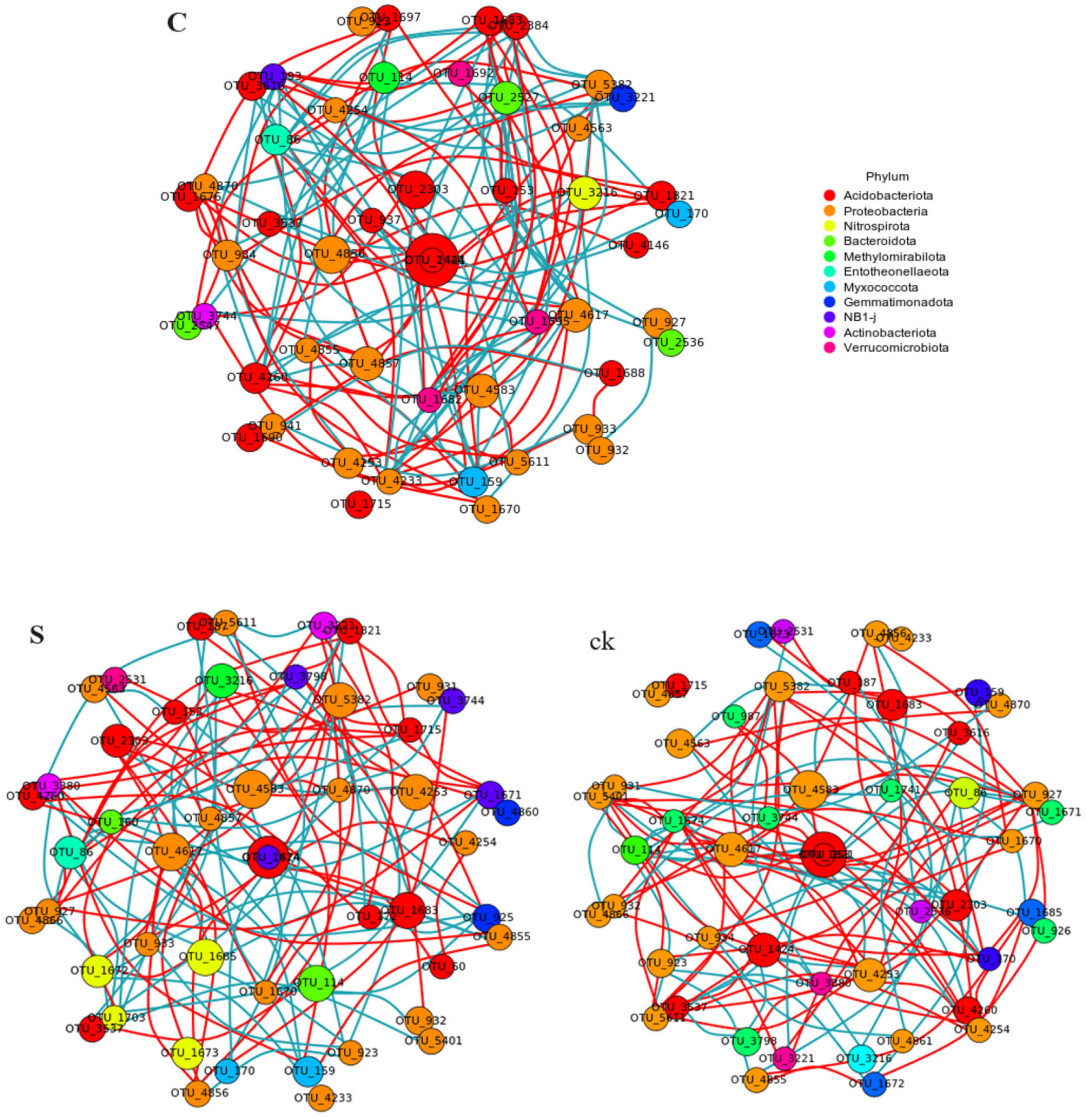
Co-occurrence network analysis of the soil rhizosphere samples revealed that the nodes in the network corresponded to the 50 most abundant OTUs, with the node sizes adjusted according to their relative abundance. Edges in red and green represent positive and negative correlations between bacteria, respectively (Pearson, *p* < 0.05). C represents teak forest intercropped with *Alpinia katsumadai* Hayata, S represents teak forest intercropped with *Amomum longiligulare* T.L. Wu, and ck represents non-intercropped teak forest.

An examination of the topological characteristics of the soil bacterial community network constructed using Spearman correlation analysis revealed that intercropping practices altered the interactions among the bacterial groups (Figure 5; Table 2). The C, S, and ck soil samples contained 106, 123, and 102 edges, respectively. Similarly, the numbers of nodes were 92, 92, and 90, respectively. Notably, the proportion of positive interactions increased, and the proportion of negative interactions decreased following intercropping. Among the samples, S exhibited the highest network density and modularity, suggesting a more intricate bacterial community structure. Conversely, ck displayed a lower network density, with approximately half of its edges showing negative connections, indicating a less complex bacterial community organization. The hubs (highly connected nodes, i.e., degrees) of S belonged to *Acidobacteriota, Actinobacteriota*, and *Proteobacteria*. *Methylomirabilota* and *Myxococcota*, respectively. *Acidobacteriota* and *Proteobacteria* in C and *Proteobacteria* and *Acidobacteriota* in ck were the central groups associated with the soil response.

**Table 2:** Topological characteristics of rhizosphere soil bacterial networks under different intercropping systems.

	**Nodes**	**Edges**	**Positive edge proportion**	**Negative edge proportion**	**Network density**	**Average degree**	**Modularity**
C	92	106	51.96%	48.04%	0.026	2.217	0.697
S	92	123	56.60%	43.40%	0.029	2.674	0.828
ck	90	102	50.41%	49.59%	0.024	2.356	0.668

C represents teak forest intercropped with *Alpinia katsumadai* Hayata, S represents teak forest intercropped with *Amomum longiligulare* T.L. Wu, and ck represents non-intercropped teak forest.

### 3.5 Relationships between soil properties and the soil bacterial community

**Figure 6:**
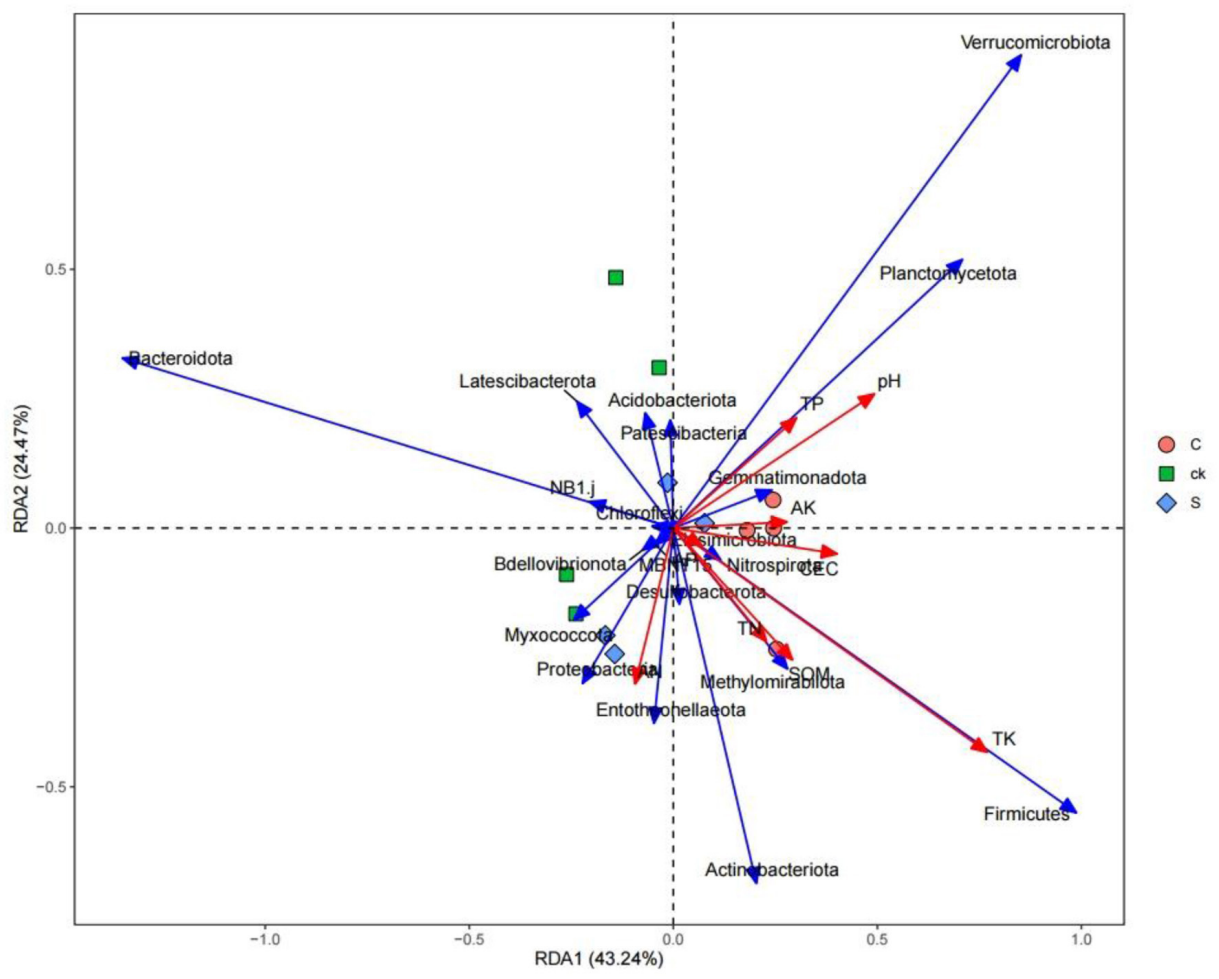
RDA of rhizosphere soil properties and bacterial community structure.

Redundancy analysis between rhizosphere soil bacterial communities and soil properties revealed that soil properties accounted for 67.71% of the total variation at the phylum level (Figure 6). Soil total potassium (TK) had the greatest influence on the bacterial community composition, followed by the soil pH.

Spearman correlation analysis of the rhizosphere soil samples with respect to the top 20 bacterial abundances (phylum level) and soil properties revealed that the relative abundance of *Planctomycetota* was significantly positively correlated with soil pH (Figure 8). The relative abundance of *Bdellovibrionota* was significantly positively correlated with SOM, TN, and AN. The relative abundance of *Latescibacterota* was negatively correlated with TP and AN. The relative abundance of *MBNT15* was negatively correlated with soil AP and AK (*p* < 0.01). The relative abundances of *Bacteroidota* and *NB1_j* were significantly negatively correlated with soil TK (*p* < 0.01), while the relative abundances of *Methylomirabilota* and *Firmicutes* were significantly positively correlated with soil TK (*p* < 0.01).

## 4 Discussion

### 4.1 Effects of intercropping on soil properties

Understory intercropping can enhance shade conditions, prevent summer heat damage, and improve soil properties (Liang et al., 2019). In this study, the soil properties of forest areas intercropped with medicinal plants and forest areas without intercropping (ck) were compared. As a crucial index of soil quality and fertility, SOM has a significant impact on ecosystem productivity (Liang et al., 2017). The results of this study revealed that medicinal plant intercropping in forests was beneficial for increasing SOM (Table 1). This difference may be attributed to two factors. First, intercropping increases the production of surface litter, which decomposes into soil organic matter (Zhang et al., 2016), second, intercropping stimulates root growth and the secretion of substances that indirectly enrich organic matter (Gu et al., 2016). Notably, intercropping significantly increased the levels of TK and AN, suggesting that the circulation and turnover of these nutrients increased, as reported in other studies (Manici et al., 2015).

Intercropping led to an increase in the soil pH, bringing it closer to neutrality than did non-intercropping (Table 1). Notably, teak prefers to grow in neutral or slightly alkaline soils (Wehr et al., 2017), suggesting that intercropping leads to conditions conducive to teak growth compared to non-intercropped forests. The increase in pH might be linked to precipitation patterns. Intercropping increases soil and water conservation, thereby preventing soil nutrients from being washed away and leading to acidity due to the absence of alkali ions (Yu et al., 2021a). Furthermore, the increase in SOM content resulting from intercropping and its subsequent decomposition contributed to an increase in the soil pH (Hu et al., 2018).

### 4.2 Rhizosphere bacterial community structure under intercropping

Bacteria play a pivotal role in maintaining the stability and sustainability of forest soil ecosystems (Xue et al., 2020). Bacterial diversity serves as a crucial indicator reflecting soil health status and functionality (Srivastava et al., 2012), with any alterations potentially impacting the stability of the soil microbial community (Xu et al., 2022). Prior research has indicated that soil microbial communities are influenced by factors such as plant genotype, location, planting pattern, and growth duration (Jin et al., 2017; Fan et al., 2019). However, our findings suggest that intercropping does not significantly influence bacterial community diversity, echoing the findings of previous studies (Diakhaté et al., 2016; Chen et al., 2021). One plausible explanation is that variations in microbial community structure might not necessarily lead to changes in species diversity due to compensatory effects occurring among different taxa. Species richness may exhibit limited responsiveness to environmental shifts, often manifesting at lower taxonomic levels rather than across the entire community (Li et al., 2021).

Although there was no significant difference in bacterial community diversity, our study revealed a distinct separation in soil bacterial community structure with intercropping (Figure 3). A unique rhizosphere bacterial community structure emerged when teak was intercropped with medicinal plants, highlighting the strong influence of intercropping on the rhizosphere. The impact of plant roots on the bacterial composition has been widely demonstrated in numerous species (Augusto et al., 2015; Edwards et al., 2015; Liu et al., 2019). Notably, the compositional differences in soil bacteria under intercropping were primarily driven by Bacteroidota, Actinobacteriota, Verrucomicrobiota, and Firmicutes (Figure 6), specific root secretions may have also influenced this result (Liu H. et al., 2020). Intercropping has the potential to modify soil microorganisms by influencing nutrient competition among plants and mediating the impact of root exudations (Guo et al., 2019; Ding et al., 2022).

**Figure 7:**
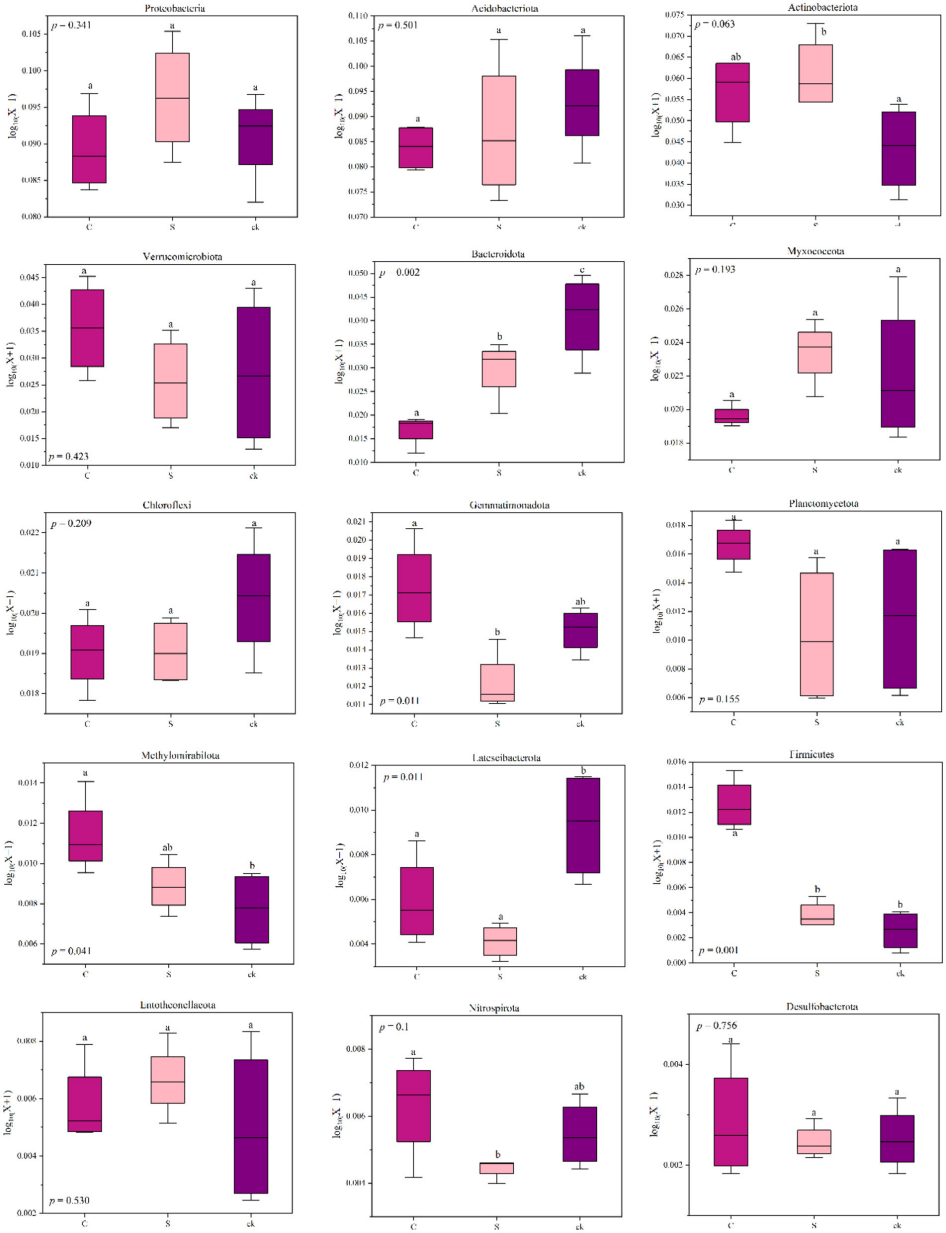
Changes in the relative abundance of bacterial phyla in different soil rhizosphere samples; distinct lowercase letters indicate notable differences (*p* < 0.05). C represents teak forest intercropped with *Alpinia katsumadai* Hayata, S represents teak forest intercropped with *Amomum longiligulare* T.L. Wu, and ck represents non-intercropped teak forest.

In general, soil microbes can adapt to changing environmental conditions by adjusting their corresponding composition and structure (Hu et al., 2019; Cao et al., 2021). In the intercropping system of teak and medicinal plants, we observed relatively high abundances of *Actinobacteriota, Methylomirabilota, Firmicutes*, and *Entotheonellaeota* (Figure 7). This high abundances may be attributed to the ecological roles of those species in organic matter degradation (Liu R. et al., 2020). Our findings revealed a significant positive correlation between the relative abundance of *Firmicutes* and soil total potassium level. This bacterial group might play a crucial role in utilizing recalcitrant carbon sources (Verzeaux et al., 2016), and it contains the highest number of diazotrophic species (Dos Santos et al., 2012). The ability of *Firmicutes* to degrade carbon and fix nitrogen has been reported (Su et al., 2015). Additionally, we observed that with increasing pH, *Verrucomicrobiota, Planctomycetota*, and other bacterial categories were significantly enriched (Figure 6), indicating a preference for a neutral environment (DeBruyn et al., 2011). When the pH falls below a certain threshold, these bacteria may lose their metabolic function (Rousk et al., 2010).

**Figure 8:**
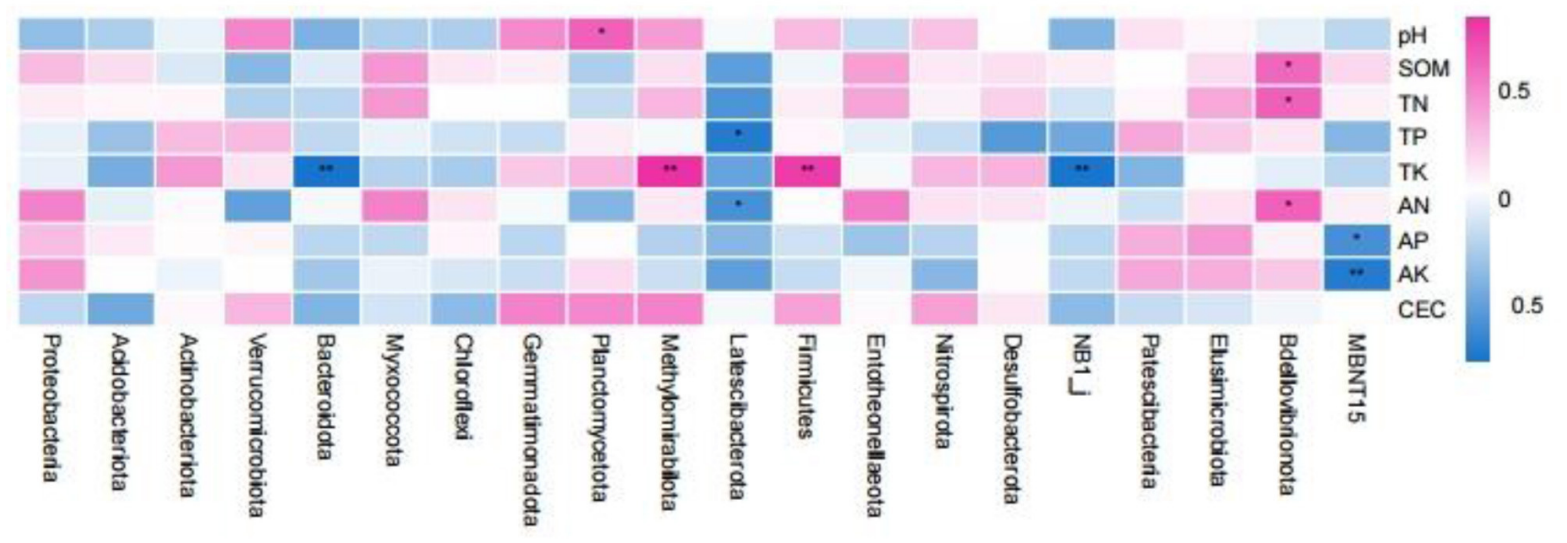
Spearman correlation analysis between the properties of the root soil samples and the bacterial communities (phylum level). *p < 0.05; **p < 0.01.

Although our study demonstrated that intercropping alters the structure of soil bacterial communities, it remains to be determined whether these changes in bacterial community composition also influence the growth of teak trees. Transplantation of rhizospheric microbiomes is a suitable approach for investigating their potential impact (Jin et al., 2023), and further studies are needed to address this question.

### 4.3 Intercropping of forest and medicine promotes a compact bacterial network structure

Previous studies have highlighted the close relationship between bacterial interactions and ecological functions, particularly in nutrient cycling and metabolism (Banerjee et al., 2016). Correlation network analysis offers a novel approach for understanding the aggregation patterns of microbial communities, reflecting ecological processes and interactions among microbial groups (Zhang et al., 2018), this method can also reveal the shared niche spaces among community members (Wu et al., 2019). A defining characteristic of microbial co-occurrence networks is the connectivity between nodes, where positive associations suggest potential niche overlaps and cooperation, while negative associations may indicate competition within these communities (Faust and Raes, 2012). In our study, we observed that the intercropping of teak with medicinal plants had a profound impact on the symbiotic network of the bacterial community. Notably, intercropping increased the number of positive edges within the network (Figure 5; Table 2), suggesting the potential enhancement of cooperative interactions among rhizosphere soil microbial groups. Conversely, in non-intercropped plots (ck), there were more negative links, possibly indicating competition among these microbial groups for limited resources (Mau et al., 2015). Taken together, those findings underscore that intercropping promotes closer and more clustered relationships among bacteria, while in non-intercropped forests, these relationships are more dispersed. Additionally, the focal groups within each network differed, suggesting a possible realignment of species interactions within the bacterial communities (Yu et al., 2021a).

The soil bacteria networks of teak intercropped with medicinal plants exhibited greater complexity than did those of non-intercropped forest areas, with more nodes and edges and greater degrees of modularity, and network density. These findings suggests that intercropping increases the diversity and connectivity of soil microbial communities. We further observed that the co-occurrence network of S samples was more closely related to that of C samples, with better positive connections, network density, and modularity of edges than C samples. Based on these observations, we conclude that teak intercropping with *A. longiligulare* T.L. Wu leads to a more complex bacterial symbiosis, forming a tighter network structure and a more stable ecosystem (Yang et al., 2018).

## 5 Conclusions

Studies have demonstrated that compared with non-intercropping, intercropping with *A. katsumadai* Hayata and *A. longiligulare* T.L. Wu increases the soil nutrient content of teak, resulting in a corresponding shift in the structure and composition of soil bacterial communities. Notably, bacterial taxa such as *Firmicutes* and *Methylomirabilota* were relatively enriched in the intercropped teak forest areas. The soil bacterial community structure appears to be influenced by soil properties such as total potassium and pH. Moreover, among the two intercropping systems, the soil bacterial structure associated with teak intercropped with *A. longiligulare* T.L. Wu was relatively more complex, suggesting a denser rhizosphere bacterial network environment. Our findings offer valuable insights for the design of teak intercropping and management systems with the aim of enhancing teak plantation productivity.

## Data availability statement

The datasets presented in this study can be found in online repositories. The names of the repository/repositories and accession number(s) can be found below: NCBI SRA, accession PRJNA1032901.

## Author contributions

WX: Investigation, Writing – original draft, Writing – review & editing. LM: Investigation, Writing – original draft. LK: Investigation, Writing – original draft. HQ: Writing – original draft. PD: Writing – original draft. MH: Writing – original draft. HG: Project administration, Writing – review & editing.
