# Supplementary material for: Effects of intercropping teak with Alpinia katsumadai Hayata and Amomum longiligulare T.L. Wu on rhizosphere soil nutrients and bacterial community diversity, structure, and network
Source: Front Microbiol. 2024 Feb 19;15:1328772. doi: 10.3389/fmicb.2024.1328772 (PMC10910098; doi:10.3389/fmicb.2024.1328772)
Supplement: Supplementary file 1 [file Data_Sheet_1.PDF]

Table S1 Statistics of Fastq data splicing results

SampleID	raw_tags	clean_tags
C1	131526	120849
C2	132445	124993
C3	119292	111242
C4	49911	48032
ck1	76239	72786
ck2	31438	30415
ck3	85899	82590
ck4	32069	31213
S1	42920	41405
S2	37803	36245
S3	64477	61238
S4	60816	58153

Note: raw_tags is the result obtained after quality control splicing of Fastq data, and clean_tags is the result obtained after further removal of chimeric and short sequences from the splicing results.

Table S2 Quality sequence length distribution statistics

distribute	reads
0-200	0
200-260	0
260-320	375
320-360	623
360-380	348
380-400	1330
400-420	307185
420-440	507300
440-460	1922
460-480	71
480-500	7
500-520	0
520-540	0
540-560	0
560-600	0

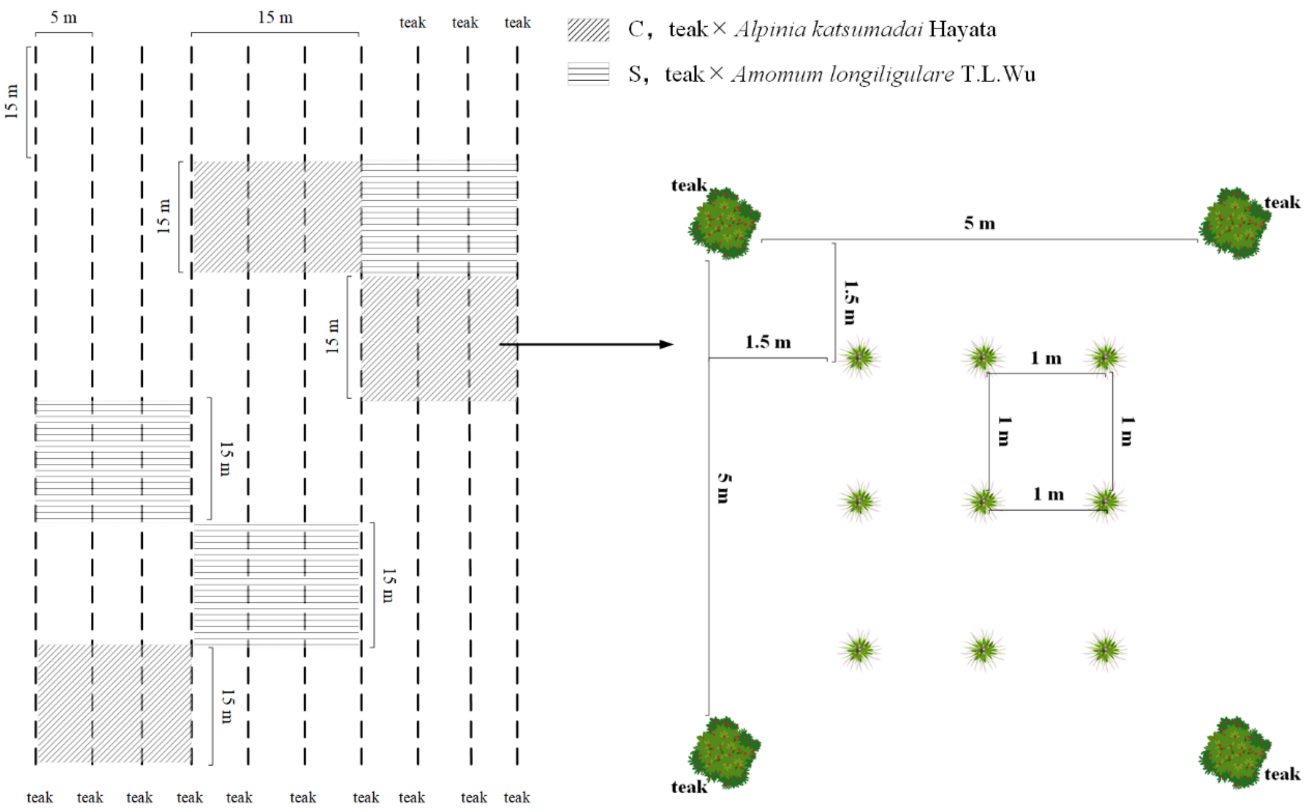

Figture S1 Interplanting test layout and sampling method diagram.

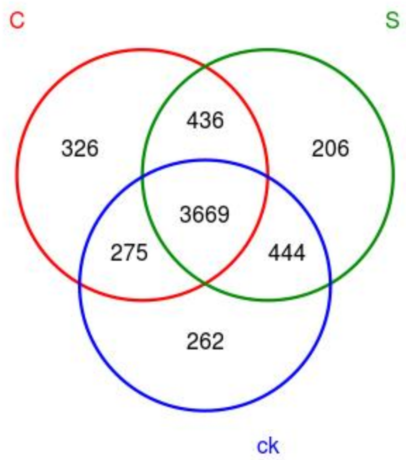

Figture S2 The number of shared and unique OTUs in soil rhizosphere samples

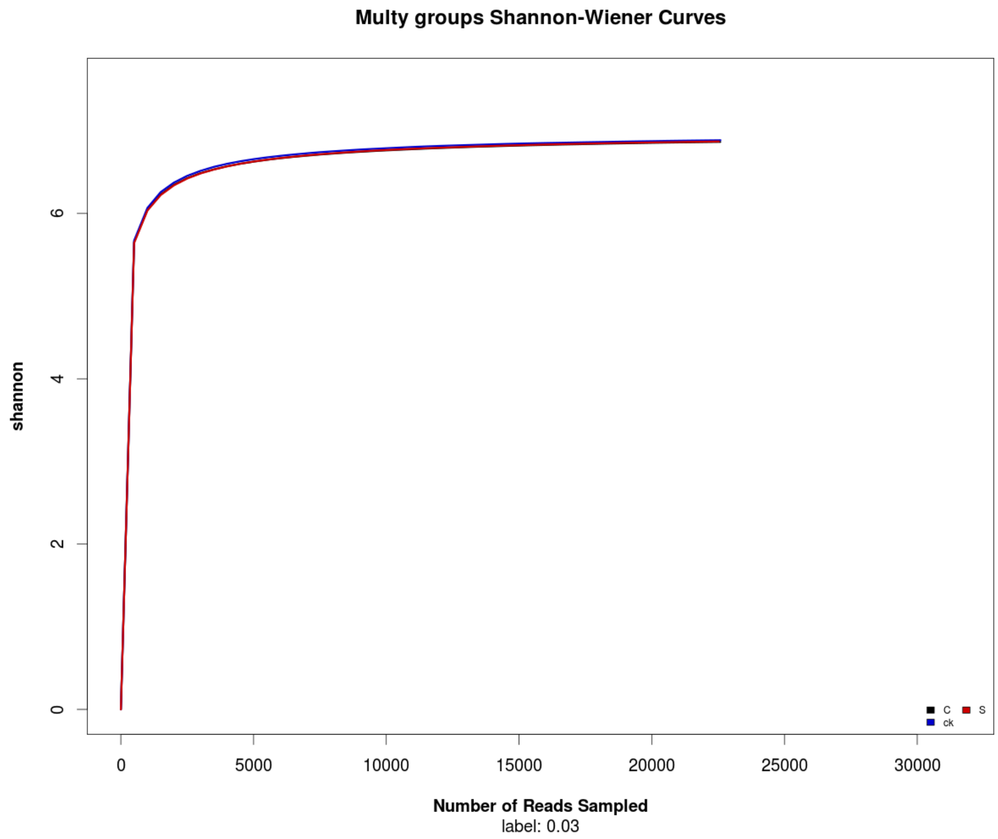

Figture S3 Rhizosphere soil samples of C, S and ck Shannon Wiener curve

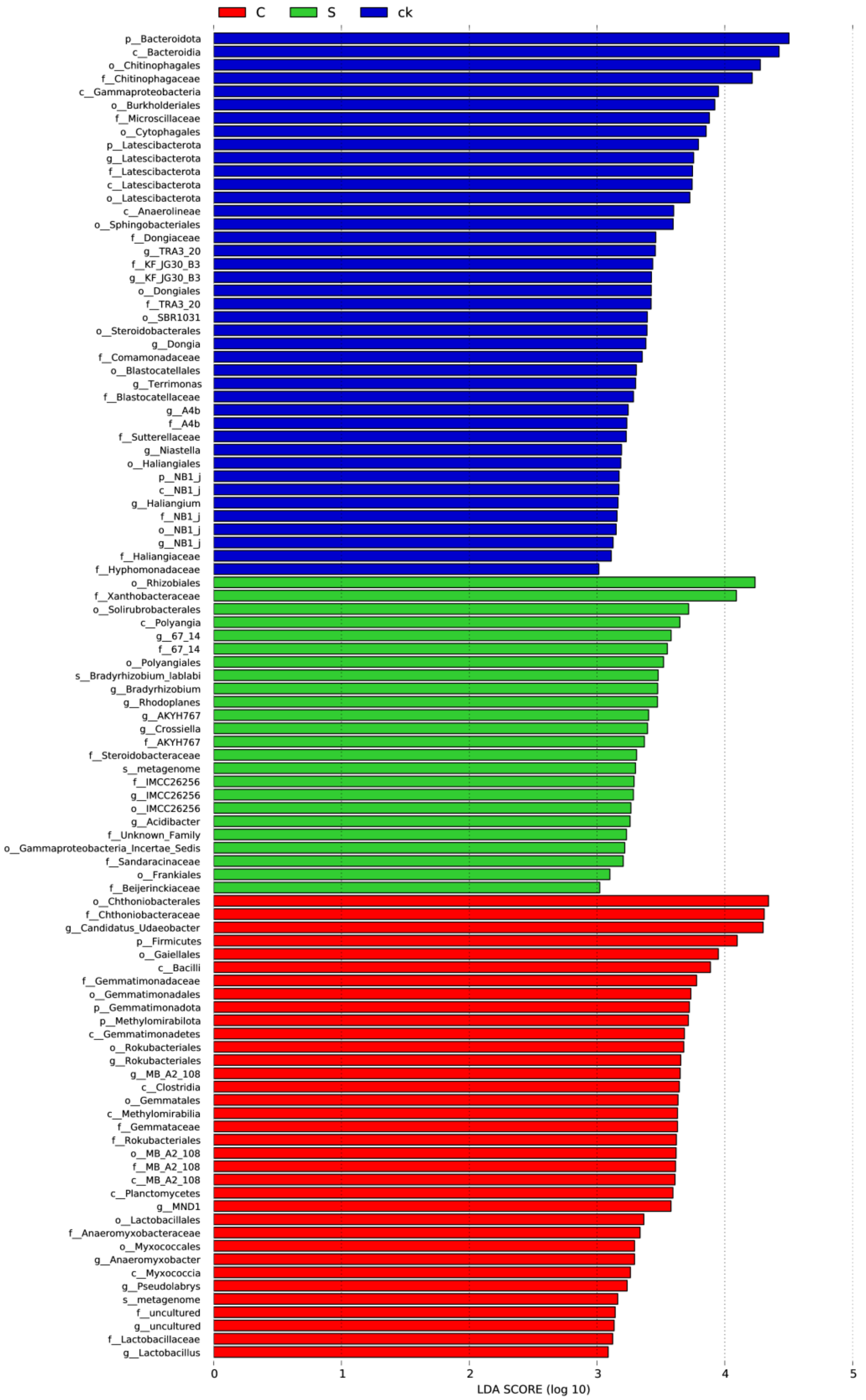

Figture S4 Linear discriminant Analysis (LDA) effect size (LEfSe) of bacterial biomarkers in rhizosphere soil samples. When the LDA score was greater than 2, the difference was statistically significant ($p < 0.05$).
